# Motoric Cognitive Risk Syndrome: Its prognostic value for dementia and other health outcomes. A systematic review

**DOI:** 10.1093/geroni/igab046.2770

**Published:** 2021-12-17

**Authors:** Donncha Mullin, Alastair Cockburn, Miles Welstead, Michelle Luciano, Graciela Muniz-Terrera, Tom Russ

**Affiliations:** 1 University of Edinburgh, Edinburgh, Scotland, United Kingdom; 2 NHS Lothian, Edinburgh, Scotland, United Kingdom; 3 University of edinburgh, Edinburgh, Scotland, United Kingdom

## Abstract

Motoric cognitive risk syndrome (MCR) is a recently defined concept combining objective slow gait and subjective cognitive complaints, in the absence of dementia or significant functional impairment. MCR is associated with an increased risk for dementia but its prognostic value for other mental and physical health conditions is less studied. MCR is quick, inexpensive and easy to measure, making it a potentially useful clinical tool. This review aims to be the first to synthesise all mental and physical health outcomes associated with MCR since the term was coined in 2013. Results from multiple databases (MEDLINE, AMED, EMBASE, CINAHL, PsycINFO, and the Cochrane library; conception to November 2020) were screened independently and risk of bias was assessed. [Results are currently preliminary but definitive findings will be available in time for the conference] In total, 1057 references were screened, resulting in 34 studies being included, of which 14 will be meta-analysed. Eleven longitudinal studies examined MCR in relation to incident cognitive impairment or dementia conversion. Other prospective studies found that MCR predicted higher risk of mortality (n=2), falls (n=3), post-fall fractures (n=1), gait dysfunction (n=1), and cardiovascular risk factors and diseases (n=1). The results from the 23 cross-sectional studies reporting associations with MCR highlight areas for further study to better understand the biological mechanisms of MCR. By synthesising the latest evidence, this review reinforces the value of MCR for predicting incident dementia, but also adds weight to its value in relation to other important age-related health outcomes.

